# Microbes and host dance in harmony or disarray?

**DOI:** 10.1007/s13238-018-0545-4

**Published:** 2018-05-02

**Authors:** Zhihua Liu

**Affiliations:** CAS Key Laboratory of Infection and Immunity, CAS Center for Excellence in Biomacromolecules, Institute of Biophysics, Chinese Academy of Sciences, Beijing, 100101 China

We are not alone. Trillion of microbes (bacteria, fungi, virus and protozoa) take residence on and in our bodies. Despite their large number, their roles in our health had been largely ignored until recently. Advances in sequencing technologies have helped us to realize the diversity and dynamics of microbes associated with our bodies. A number of international microbiome consortium programs have suggested connections between dysbiosis and a range of human diseases, including metabolic syndrome, autoimmune diseases, autism and many others. Furthermore, use of antibiotics has been found to reduce the efficacy of cancer immunotherapies. Currently, the microbiome is attracting enormous research interest internationally and has become a frontier in life sciences.

Studies have suggested that commensal microbes and the host have evolved reciprocal crosstalk to establish mutualistic relationships. For instance, the existence of vast numbers of intestinal microbes provides an enormous pool of metabolic enzymes, which can break down otherwise indigestible carbohydrates. The availability of such metabolic enzymes is subjected to flux in response to diet. A study in Tanzanian Hadza people provides an elegant example of how the microbiome changes in response to diet to optimize nutrient uptake for the host (Smits et al., 2017). The microbiome in the Hadza is highly seasonal, changing in a cycle throughout the year. The annual changes in the gut microbiome are probably caused by cyclical shifts in the Hadza diet. During the dry season, the Hadza eat a lot of meat plus tubers and fruit from the baobab tree, but in the wet season they eat more honey and berries. *Prevotella* species become predominant in the dry season, because they are particularly good at breaking down plant material. Furthermore, several studies have found that childhood malnutrition is associated with microbiota immaturity (Subramanian et al., 2014; Blanton et al., 2016). How is the microbiota established in a newborn, and how does it subsequently mature? It turns out that around 10 percent of human milk comprises complex sugars called human milk oligosaccharides (HMOs) (Charbonneau et al., 2016), which babies cannot digest. Instead, these sugars have evolved to feed a baby’s first microbes. They are the means through which mothers ensure that their newborns are colonized by beneficial bacteria. More studies have revealed how intestinal microbes profoundly shape host metabolism. In addition, commensal microbes play a critical role in host defense against pathogens by directly combating invading pathogens or educating the immune systems. Overall changes in lifestyle, such as western diet, high hygiene standards and overuse of antibiotics, have profoundly altered our microbiome composition. For example, one study on microbiota in people from Amazonas in Venezuela, rural Malawi and US metropolitan areas showed pronounced differences in bacterial assemblages and functional gene repertoires between US residents and those in the other two countries (Yatsunenko et al., 2012). Dysbiosis has been observed in syndromes associated with metabolic and immune regulation, which suggests a scenario in which altered crosstalk between host and microbes underlies the pathogenesis of the related diseases.

A few key research areas of microbiome are covered in this special issue of Protein & Cell, organized by Dr. Jun Wang from Institute of Microbiology and Dr. Fangqing Zhao from Beijing Institute of Life Science, Beijing, Chinese Academy of Sciences, including basic mechanistic studies toward understanding the host-microbial reciprocal relationship, technology developments in microbiome, therapeutics and related bioethics. Dr. Wang discusses the influence of host genetics on shaping microbiome (Wang et al., 2018), and Dr. Chen discusses the role of oral microbiome in health (Gao et al., 2018). Dr. Yu discusses the late progress on diet, microbiota and colorectal cancer (Yang and Yu, 2018). Dr. Zhao discusses the application of single-cell metagenomics in microbiome research and challenges (Xu and Zhao, 2018). Dr. Fu discusses pharcomicrobiomics and implication in personalized medicine (Doestzada et al., 2018). Dr. Zhang reviews the current concept and methodology on microbiota transplantation (Zhang et al., 2018). Last but not less, Dr. Ma discusses the ethics associated with microbiota transplantation (Ma et al., 2018).

Together, though at an early stage in understanding the profound roles of commensal microbes in our health and disease, we are thrilled at the possibilities to manipulate microbiota to boost our health. To reach this ultimate goal, we will need to understand the basics about the crosstalk between host and microbes, and how this crosstalk may go awry to promote dysbiosis-associated diseases. We will also need to understand the rules governing how the host and microbiota reach a homeostatic balance. With that information, we will be able to develop ways to manipulate the microbiota to boost health.
